# Development and Validation of a Tool to Assess the Quality of Clinical Practice Guideline Recommendations

**DOI:** 10.1001/jamanetworkopen.2020.5535

**Published:** 2020-05-27

**Authors:** Melissa C. Brouwers, Karen Spithoff, Kate Kerkvliet, Pablo Alonso-Coello, Jako Burgers, Francoise Cluzeau, Beatrice Férvers, Ian Graham, Jeremy Grimshaw, Steven Hanna, Monika Kastner, Michelle Kho, Amir Qaseem, Sharon Straus, Ivan D. Florez

**Affiliations:** 1 University of Ottawa, Ottawa, Ontario, Canada; 2 McMaster University, Hamilton, Ontario, Canada; 3 Iberoamerican Cochrane Centre, Biomedical Research Institute Sant Pau (IIB Sant Pau-CIBERESP), Barcelona, Spain; 4 Dutch College of General Practitioners, Utrecht, the Netherlands; 5 Imperial College London, St Mary’s Hospital, London, United Kingdom; 6 Département Cancer et Environnement, Centre Léon Bérard, Lyon Cedex 08, France; 7 Ottawa Hospital Research Institute, University of Ottawa, Ottawa, Ontario, Canada; 8 North York General Hospital, Toronto, Ontario, Canada; 9 Institute of Applied Health Sciences, McMaster University, Hamilton, Ontario, Canada; 10 American College of Physicians, Philadelphia, Pennsylvania; 11 Li Ka Shing Knowledge Institute of St. Michael's Hospital, Toronto, Ontario, Canada; 12 Department of Pediatrics, University of Antioquia, Medellín, Colombia

## Abstract

**Importance:**

Clinical practice guidelines (CPGs) may lack rigor and suitability to the setting in which they are to be applied. Methods to yield clinical practice guideline recommendations that are credible and implementable remain to be determined.

**Objective:**

To describe the development of AGREE-REX (Appraisal of Guidelines Research and Evaluation–Recommendations Excellence), a tool designed to evaluate the quality of clinical practice guideline recommendations.

**Design, Setting, and Participants:**

A cross-sectional study of 322 international stakeholders representing CPG developers, users, and researchers was conducted between December 2015 and March 2019. Advertisements to participate were distributed through professional organizations as well as through the AGREE Enterprise social media accounts and their registered users.

**Exposures:**

Between 2015 and 2017, participants appraised 1 of 161 CPGs using the Draft AGREE-REX tool and completed the AGREE-REX Usability Survey.

**Main Outcomes and Measures:**

Usability and measurement properties of the tool were assessed with 7-point scales (1 indicating strong disagreement and 7 indicating strong agreement). Internal consistency of items was assessed with the Cronbach α, and the Spearman-Brown reliability adjustment was used to calculate reliability for 2 to 5 raters.

**Results:**

A total of 322 participants (202 female participants [62.7%]; 83 aged 40-49 years [25.8%]) rated the survey items (on a 7-point scale). All 11 items were rated as easy to understand (with a mean [SD] ranging from 5.2 [1.38] for the alignment of values item to 6.3 [0.87] for the evidence item) and easy to apply (with a mean [SD] ranging from 4.8 [1.49] for the alignment of values item to 6.1 [1.07] for the evidence item). Participants provided favorable feedback on the tool’s instructions, which were considered clear (mean [SD], 5.8 [1.06]), helpful (mean [SD], 5.9 [1.00]), and complete (mean [SD], 5.8 [1.11]). Participants considered the tool easy to use (mean [SD], 5.4 [1.32]) and thought that it added value to the guideline enterprise (mean [SD], 5.9 [1.13]). Internal consistency of the items was high (Cronbach α = 0.94). Positive correlations were found between the overall AGREE-REX score and the implementability score (*r* = 0.81) and the clinical credibility score (*r* = 0.76).

**Conclusions and Relevance:**

This cross-sectional study found that the AGREE-REX tool can be useful in evaluating CPG recommendations, differentiating among them, and identifying those that are clinically credible and implementable for practicing health professionals and decision makers who use recommendations to inform clinical policy.

## Key Points

**Question:**

Is it possible to create a tool to specifically evaluate the quality of clinical practice guideline recommendations?

**Findings:**

In this cross-sectional study of 322 international stakeholders, the Appraisal of Guidelines Research and Evaluation–Recommendations Excellence (AGREE-REX) tool was developed to appraise guidelines for clinical practice. All participants rated the tool as usable and agreed that it represents a valuable addition to the clinical practice guidelines enterprise.

**Meaning:**

A panel of stakeholders agrees that the AGREE-REX tool may provide information about the methodologic quality of guideline recommendations and may help in the implementation of clinical practice guidelines.

## Teaser

This cross-sectional study examines a tool for assessing the credibility and implementability of clinical practice guidelines.

## Introduction

Clinical practice guidelines (CPGs) are systematically developed statements informed by a systematic review of evidence and an assessment of the benefits and harms of care options designed to optimize patient care.^1,2,3^ The potential benefits of CPGs, however, are only as good as their quality. Appropriate methods and rigorous development strategies are important factors in the successful implementation of CPG recommendations.^4,5,6,7,8,9,10^ Not all CPGs are alike; their quality is variable and often falls short of reported goals.^11,12,13,14,15,16,17,18,19^

The Appraisal of Guidelines, Research and Evaluation revision (AGREE II) tool has become an accepted international resource to evaluate the quality of CPGs and to provide a methodologic framework to inform CPG development, reporting, and evaluation.^5,6,7,20,21,22^ The AGREE II tool targets the entire CPG development process and all components of the CPG report: the articulation of scope and practice, who is involved, methods used, applicability, editorial independence, and clarity.

Since the release of AGREE II, studies have reported that high AGREE II scores do not guarantee that the resulting CPG recommendations are optimal.^23,24,25,26,27^ For example, Nuckols et al^24^ evaluated the technical quality and acceptability of 5 musculoskeletal CPGs. Use of the AGREE II tool resulted in high quality scores (eg, rigor domain scores >80%). However, participants reported that the CPGs omitted common clinical situations and contained recommendations of uncertain clinical validity. Similar results have been found with disability-related CPGs.^26^

These studies suggest that a distinction exists between user perceptions of a CPG report and the report’s recommendations. Hence, a barrier may exist if users rely solely on the AGREE II quality scores in making decisions about which CPG recommendations to implement or which CPGs to adapt to a specific context. For example, if a CPG provides insufficient information about the values of patients, health care professionals, and funders, or there is a lack of alignment across different viewpoints, that CPG may yield recommendations that are difficult to use and implement, even if the evidence base is solid or the methods used to create the CPG are of high quality. The CPGs that address controversial issues in which values clash (eg, medically assisted dying) may be especially susceptible to this concern. Inadequate consideration of different perspectives and varied implementation concerns are a common limitation in CPG appraisal tools.^28^

The development of AGREE II focused primarily on methodologic quality and internal validity of the CPG report and to a lesser extent on the external validity of the recommendations. A more thorough investigation of the implementation science literature and the usability and relevance of recommendations was warranted. Our international team of CPG developers and researchers created the AGREE-REX (Appraisal of Guidelines Research and Evaluation–Recommendations Excellence) tool to evaluate the quality of CPG recommendations specifically, defined as credible and implementable recommendations.

## Methods

### Development of Draft AGREE-REX

The development process used international standards of measurement design.^29^ Our first step required identification of candidate items. This step was completed and is described in previous studies.^30,31^ In brief, a realist review was conducted to identify attributes of CPGs associated with the implementation of their recommendations. The review resulted in the Guideline Implementability for Decision Excellence Model (GUIDE-M) that was vetted by the international CPG community.^30^ This multilevel model comprises 3 core tactics, 7 domains, and approximately 100 embedded components. The model was evaluated by 248 stakeholders from 34 countries and refined.

A core domain of the model (deliberations and contextualization) provided content coverage of our concept of CPG recommendation quality. The domain is composed of 3 subdomains, 11 attributes, and many subattributes and elements: clinical applicability (clinical, patient, and implementability relevance), values (perspectives of patient, health care professional, population, policy, developer), and feasibility (local, novelty, resources).

We derived candidate items from these data that 15 international CPG stakeholders evaluated. We used this feedback to refine the content and create the Draft AGREE-REX, used in this study (eAppendix 2 in the Supplement). The Draft AGREE-REX comprises 11 items (4 themes) and 2 overall items.

Three response scales were designed to rate each item of the Draft AGREE-REX. Two mandatory 7-point response scales (with 1 indicating strongly disagree and 7 indicating strongly agree) asked appraisers to rate the extent to which quality criteria are reported in the CPG (documentation scale) and then used to inform the CPG recommendations (consideration scale). An optional 7-point scale asked appraisers whether the documented and considered information aligned with, and was suitable for use in, their context (suitability scale). This scale was designed for use only when CPG recommendations from an authoring group are being considered for endorsement, adaptation, or implementation by another group. Two overall items asked appraisers for their overall ratings of the implementability of the CPG recommendations and their overall ratings of the clinical credibility of the CPG recommendations. Each item was answered according to a 7-point scale.

### Participants

To test the Draft AGREE-REX tool, a cross-sectional study design was used. The CPG users, developers, researchers, or trainees were eligible to participate. Between December 2015 and March 2017, advertisements to participate were distributed through professional organizations (eg, the Guidelines International Network) as well as through the AGREE Enterprise social media accounts and their registered users. Given the nature of the recruitment strategy and the substantial number of cross-postings, an accurate number of individuals the advertisements reached is not available. Completion of the study implied consent and participants were offered a CAD$50 gift card. The study received ethics approval from the Hamilton Integrated Research Ethics Board.

The CPGs were selected from the National Guideline Clearinghouse of the Agency for Healthcare Research and Quality. Selection criteria were as follows: English language, published between 2013 and 2015, and length of core CPG document less than 50 pages.

The target sample size was calculated based on the interrater reliability outcome, assuming 2 raters per CPG, an intraclass correlation coefficient of 0.6, and a CI from 0.5 to 0.7. On the basis of these assumptions, 316 participants were required to appraise 158 CPGs. This study followed the Strengthening the Reporting of Observational Studies in Epidemiology (STROBE) reporting guideline for cross-sectional studies.

### Procedures

Participants were required to read a single CPG, evaluate the entire set of recommendations with the Draft AGREE-REX, and complete the AGREE-REX Usability Survey. Individuals who responded to the advertisement were sent an email with an invitation letter, an electronic copy of the Draft AGREE-REX, the CPG to which they were randomly assigned, and access to LimeSurvey to submit AGREE-REX appraisal scores and to complete the AGREE-REX Usability Survey. Reminder emails were sent to nonrespondents at 2-week intervals up to 3 times.

Using the three 7-point scales, participants were asked to rate the items, the instructions, the response scale, their ability to apply the tool, and its usefulness. For each Draft AGREE-REX item, ratings from the documentation scale and the considerations scale were calculated as a mean between the 2 appraisers. Strong positive correlations between the 2 rating scales emerged (defined as an *r* >0.90), and analyses produced identical patterns of results.

An overall AGREE-REX score was calculated by adding the mean item scores from the consideration scale and scaling the total as a percentage of the maximum possible score. These scores were used to assess the tool’s measurement properties. The AGREE-REX ratings of the CPGs appraised in the study have been reported.^30^

Two research staff members (K.S and K.K) with formal training and experience independently evaluated all the CPGs with the AGREE II tool. The AGREE II tool comprises 23 items within 6 domains. Each item is answered using a 7-point agreement scale with higher ratings indicating higher CPG quality.^5^ The AGREE II domain scores were used as part of the analytical framework to assess the performance of the Draft AGREE-REX.

### Statistical Analysis

Quantitative data were analyzed using SPSS software, version 24 (IBM Corp). Means and SDs for each of the items in the AGREE-REX Usability Survey were calculated. Cronbach α and correlations-if-item-deleted were calculated to assess the internal consistency of the items. Intraclass correlations were calculated for 2 to 5 appraisers using the Spearman-Brown reliability adjustment to assess the reliability of the overall AGREE-REX score.^29,32,33^ A 2-tailed *P* < .05 was considered as statistically significant.

Differentiating itself from the AGREE II tool, the AGREE-REX tool evaluates the quality of CPG recommendations, defined as the extent to which they are credible and implementable. Thus, to explore construct validity, correlations between the overall AGREE-REX score and the implementability score and the clinical credibility score were calculated, with the expectation that positive correlations would emerge. As an exploratory measure of discriminant validation, the correlations between the overall AGREE-REX score and AGREE II domain scores, assuming the mean scores across 4 raters and correcting for the attenuation in the correlation due to measurement error, were also calculated. The correlations of the former were expected to be larger than those of the latter. No standard for CPG recommendation quality currently exists; thus measures of criterion validity were not appropriate.^23,32,33^

Participants provided written feedback, and themes that emerged were noted. Formal thematic analysis was not undertaken.

Using the quantitative data and the written feedback from participants, the research team used an iterative process to refine the Draft AGREE-REX tool. This refinement was achieved through an in-person meeting, a feedback session with stakeholders at the 2017 Global Evidence Summit,^34^ and multiple teleconference meetings with the AGREE-REX team (2017-2019). Decisions were reached by consensus.

## Results

Of the 692 individuals who responded to the advertisement and were emailed a formal invitation, 322 (47.0%) completed the study. Of the 322 respondents, 202 (62.7%) were female, 252 (78.2%) had some experience with the AGREE II tool, 188 (58%) indicated that English was their first language, and 170 (53.8%) identified themselves as CPG developers (Table 1). Participants represented 6 geographic regions; 177 (55.0%) were from North America, 76 (24.0%) from Europe, 32 (10.0%) from South America, 24 (7.4%) from Asia, 7 (2.1%) from Africa, and 6 (2.0%) from Oceania.

**Table 1.** Characteristics of 322 Participants

Demographic characteristic	Frequency, No. (%)
Sex	
Female	202 (62.7)
Male	115 (35.7)
Prefer not to disclose	5 (1.6)
Age, y	
19 or younger	2 (0.6)
20-29	49 (15.2)
30-39	100 (31.1)
40-49	83 (25.8)
50-59	63 (19.6)
60-69	23 (7.1)
≥70	2 (0.6)
Experience with AGREE II	
No experience	70 (21.7)
Some experience	122 (37.9)
Experienced	88 (27.3)
Very experienced	42 (13)
First language	
English	188 (58.4)
Spanish	51 (15.8)
Italian	14 (4.3)
Chinese	13 (4)
Dutch	10 (3.1)
Portuguese	7 (2.2)
French	4 (1.2)
Greek	3 (0.9)
Ukrainian	3 (0.9)
Other	29 (9)
Geographic location	
North America	177 (55)
Europe	76 (23.6)
Asia	24 (7.5)
South America	32 (9.9)
Africa	7 (2.2)
Oceania	6 (1.9)
Participants’ role with clinical practice guidelines (as many as apply)	
Practice guideline developer	
Clinical expert	85 (26.4)
Patient/public representative	15 (4.7)
Methodologist	170 (52.8)
Practice guideline user	
Health care professional	102 (31.7)
Administrator/policy maker/manager	38 (11.8)
Patient/member of the public	20 (6.2)
Researcher	159 (49.4)
Other (eg, librarian, student)	25 (7.8)

Abbreviation: AGREE II, Appraisal of Guidelines, Research and Evaluation revision.

As reported in Table 2 and Table 3, participants rated the survey items as easy to understand (with a mean [SD] ranging from 5.2 [1.38] for the alignment of values item to 6.3 [0.87] for the evidence item on the 7-point scale) and easy to apply (with a mean [SD] ranging from 4.8 [1.49] for the alignment of values item to 6.1 [1.07] for the evidence item on the 7-point scale). Participants rated the tool’s instructions on the 7-point scale as clear (mean [SD], 5.8 [1.06]), felt confident in applying the tool to a guideline (mean [SD], 5.1 [1.43]), regarded the tool as complete (mean [SD], 5.7 [1.18]), and agreed that the tool adds value to the CPG enterprise (mean [SD], 5.9 [1.13]). In addition, 229 (71%) of respondents intended to use the AGREE-REX tool for evaluation, 203 (63%) for endorsement, and 187 (58%) for development or reporting purposes.

**Table 2.** AGREE-REX Section 1 Usability Survey Results From 322 Participants

Section 1 item^a^	Participant rating, mean (SD)	
	Easy to understand	Easy to apply
Evidence	6.3 (0.87)	6.1 (1.07)
Clinical relevance	6.2 (0.80)	5.9 (1.06)
Relevance to patients/populations	6.1 (0.89)	5.8 (1.07)
Implementation relevance	5.8 (0.99)	5.4 (1.31)
Guideline developer values	5.6 (1.20)	5.2 (1.37)
Target user values	5.7 (1.20)	5.3 (1.37)
Patient or population values	5.7 (1.15)	5.3 (1.35)
Policy values	5.4 (1.26)	5.1 (1.41)
Alignment of values	5.2 (1.38)	4.8 (1.49)
Local applicability	5.9 (1.05)	5.4 (1.33)
Resources, capacity and tools	6.0 (0.96)	5.6 (1.28)

Abbreviation: AGREE-REX, Appraisal of Guidelines for Research and Evaluation–Recommendations Excellence.

^a^ From Section 1 of the survey: asks agreement, with a response of 1 indicating strongly disagree and 7 indicating strongly agree.

**Table 3.** AGREE-REX Section 2 Usability Survey Results From 322 Participants

Section 2 item^a^	Participant rating, mean (SD)
The AGREE-REX instructions are clear	5.8 (1.06)
The AGREE-REX instructions are helpful	5.9 (1.00)
The AGREE-REX instructions are complete	5.8 (1.11)
The AGREE-REX was easy to use	5.4 (1.32)
I felt confident when applying the AGREE-REX to a guideline	5.1 (1.43)
The AGREE-REX is complete; there are no missing items	5.7 (1.18)
The use of multiple evaluation statements for each of the 11 items is appropriate	5.5 (1.52)
The use of a 7-point response scale is appropriate	5.9 (1.28)
The overall assessment questions are useful	5.9 (1.06)
The AGREE-REX would be useful for	
Evaluating a guideline	5.8 (1.29)
Guideline development and reporting	6.0 (1.19)
Deciding whether or not to adapt or endorse a guideline	5.7 (1.27)
Deciding whether or not to implement a guideline in clinical practice	5.7 (1.25)
The AGREE-REX adds value to the clinical practice guideline enterprise	5.9 (1.13)

Abbreviation: AGREE-REX, Appraisal of Guidelines for Research and Evaluation–Recommendations Excellence.

^a^ From Section 2 of the survey: asks agreement, with a response of 1 indicating strongly disagree and 7 indicating strongly agree.

Internal consistency of the items was high (Cronbach α = 0.94); deleting an item did not alter this finding. Interrater reliability predicted for the mean of 2 was 0.47, of 3 was 0.57, of 4 was 0.64, and of 5 was 0.69.

Correlation between the overall AGREE-REX score and the implementability score was 0.81 and between the overall AGREE-REX score and the clinical credibility score was 0.76 and more robust than the correlations between the overall AGREE-REX score and each of the AGREE II domain scores (for example, *r* = 0.10 for clarity of presentation and *r* = 0.43 for applicability) (Table 4).

**Table 4.** Correlations Between 161 Guidelines

Variable	Overall AGREE-REX score	
	Pearson *r*	*P* value
AGREE II domain score		
1. Scope and purpose	0.25	<.001
2. Stakeholder involvement	0.29	<.001
3. Rigor of development	0.27	.001
4. Clarity of presentation	0.10	.23
5. Applicability	0.43	<.001
6. Editorial independence	0.12	.12
AGREE-REX item score		
Overall implementability score	0.81	<.001
Overall clinical credibility score	0.76	<.001

Abbreviation: AGREE-REX, Appraisal of Guidelines for Research and Evaluation–Recommendations Excellence.

Participants offered wording changes and editorial suggestions to help clarify concepts and ideas. Core themes emerged in the written feedback. For Draft AGREE-REX and AGREE II, some participants articulated concerns about how to use both tools, potential redundancy, and lack of instruction. Some participants preferred having the tools separate and others suggested they be integrated. For Draft AGREE-REX content and usability, participants articulated challenges in applying some items in the values theme and offered suggestions for clarity. Most participants did not like the 2 response scales or could not differentiate the intent between them.

### Final Refinements

Based on the study results and feedback from participants, changes were made to the tool. Table 5 lists the final items and criteria. eAppendix 1 in the Supplement compares the draft with the final version 1 of the tool and eAppendix 2 provides the entire AGREE-REX User’s Guide.

**Table 5.** AGREE-REX (Version 1) Items and Criteria

Item	Criteria
**Item 1. Evidence**	
Definition: To be of high quality, recommendation should be based on a thorough review of the quality and results of the available evidence^a^	The guideline assesses any risk of bias related to the study designs of the supporting evidence
	The guideline describes the consistency of the results (ie, similarity of results across studies)
	The guideline addresses the directness of the evidence (ie, addresses the exact interventions, populations, and outcomes of interest) to the clinical/health problem
	The guideline indicates the precision of the results (eg, width of confidence intervals of individual studies or meta-analyses)
	The guideline describes the magnitude of the benefits and harms
	The guideline assesses the likelihood of publication bias
	The guideline addresses the possibility of confounding factors (if applicable)
	The guideline indicates the dose-response gradient (if applicable)
**Item 2. Applicability to target users**	
This item evaluates the degree to which the recommendations are applicable to the guideline’s target users’ practice context	The guideline addresses a clinical/health problem that is relevant to the intended target user(s)
	There is an alignment between the target user’s scope of practice and targeted patients/populations
	Target user’s scope of practice and recommended actions
	The direction of the recommendations (ie, in favor of or against a particular action) and the trade-offs between harms and benefits
	The definitiveness or strength of the recommendations and the trade-offs between harms and benefits
**Item 3. Applicability to patients or populations**	
This item assesses the extent to which the anticipated outcomes of the recommended action are relevant for, and valued by, the intended patients/populations	The guideline includes outcomes that are relevant to the targeted patients/populations. These outcomes are often referred to as patient-important outcomes, patient-centered outcomes, patient-reported outcomes, or patient experience
	Relevant outcomes were considered in the development of the evidence base
	Recommended actions have the potential to affect outcomes relevant to patients/populations (eg, improve desirable patient-relevant outcomes, mitigate undesirable patient-relevant outcomes)
	The guideline reports how the importance of outcomes to patients was determined
	The guideline describes how to tailor recommendations for application to individual (or subsets of) patients or populations (eg, based on age, sex, ethnicity, comorbidities)
**Item 4. Values and preferences of target users**	
Values and preferences of target users refers to the relative importance that the target users of the guidelines (eg, health care providers, policy makers, administrators) place on the outcomes of interest (eg, survival, adverse effects, quality of life, cost, convenience). Target user values and preferences are important to consider during the guideline development process because they influence whether the recommendations are acceptable and adopted into practice	Values and preferences of guideline target users, as they relate to the recommended actions, have been sought and considered
	Factors related to target user acceptability of the recommended actions have been considered (eg, the acceptability of learning new clinical skills or the need to adapt current routine)
	The guideline differentiates between recommended actions for which clinical flexibility and individual patient tailoring are more appropriate in the decision-making process and those for which they are less appropriate
	The guideline describes the range of recommended actions that are acceptable to the clinical community, including the preferred option (if relevant), and describing why it is the preferred choice
**Item 5. Values and preferences of patients/populations**	
Values and preferences of patients/populations refers to the relative importance that the recipients of the recommended actions place on the outcomes of interest (eg, survival, adverse effects, quality of life, cost, convenience). Patient or population values and preferences are important to consider during the guideline development process because they influence whether the recommendations are acceptable and adopted into practice	The guideline includes outcomes that are relevant to the targeted patients/populations. These outcomes are often referred to as patient-important outcomes, patient-centered outcomes, patient-reported outcomes, or patient experience
	Relevant outcomes were considered in the development of the evidence base
	Recommended actions have the potential to affect outcomes relevant to patients/populations (eg, improve desirable patient-relevant outcomes, mitigate undesirable patient-relevant outcomes)
	The guideline reports how the importance of outcomes to patients was determined
	The guideline describes how to tailor recommendations for application to individual (or subsets of) patients or populations (eg, based on age, sex, ethnicity, comorbidities)
**Item 6. Values and preferences of policy/decision-makers**	
Values and preferences of policy/decision-makers refers to the relative importance that policy stakeholders place on the outcomes of interest (eg, survival, adverse effects, quality of life, cost, convenience). The values and preferences of policy stakeholders can affect the implementation of guideline recommendations in the health care system (eg, provision of resources or funding to support the recommended actions)	Information about the needs of policy and decision-makers has been sought and considered in the formulation of the recommendations
	The effect of the recommendations on policy and system-level decision-making has been considered in the formulation of the recommendations
	The effect of the recommendations on health equities has been considered in the formulation of the recommendations
	The guideline describes where changes to policy should be made to align with the recommendations
**Item 7. Values and preferences of guideline developers**	
Values and preferences of guideline developers refers to the relative importance that developers place on the outcomes of interest (eg, survival, adverse effects, quality of life, cost, convenience). Guideline developer values can influence the selection of outcomes of interest, the choice of guideline development methods, the approach to integrating varying stakeholder perspectives, and the interpretation of the balance between benefits and harms.	There is a clear description of the values and preferences that guideline developers brought to the development process
	There is a clear description of how guideline developer values and preferences influenced their interpretation of the balance between benefits and harms
	The method used to integrate values and preferences, including when they differ between stakeholders (eg, target users, patients/population, policy makers), is described
**Item 8. Purpose**	
Practice guidelines can be developed to achieve several implementation goals, such as to influence health care decisions, to promote discussion in the clinical encounter, to provide rationale to create or refine clinical policy, or to identify actions that reflect clinical or population health goals.	The guideline recommendations align with the implementation goals of the guideline (eg, for advocacy or policy change)
	The anticipated effects of recommendation adoption on individuals (eg, patients, populations, target users), organizations, and/or systems are described
**Item 9. Local application and adoption**	
This item assesses the suitability of the guideline recommendations for the setting, patients/population, and/or the health care system in which they are being implemented. Guidelines that include advice or tools and resources to facilitate the implementation of the recommendations are easier to adopt in practice.	The guideline describes the types and degree of change required from current practice
	The guideline differentiates between recommendations for which local adaptation may be more or less relevant
	The guideline articulates relevant factors important to its successful dissemination
	The guideline developers considered the issues that can influence the adoption of the recommendations and provided tools and/or advice for guideline implementers related to: How to tailor recommendations for the local setting Resource considerations needed to implement the recommendations (eg, human resources, equipment) and their associated costs Economic analysis (eg, cost-effectiveness or cost-utility) of recommended actions (if appropriate) Competencies and/or training of personnel required to implement the recommended action Data required to implement and monitor the adoption of recommended actions Strategies to overcome barriers related to health care professional acceptability and/or patient/population and/or policy acceptability of the recommended actions Criteria that can be used to measure recommendation implementation and quality improvement

Abbreviation: AGREE-REX, Appraisal of Guidelines for Research and Evaluation–Recommendations Excellence.

^a^ Informed by GRADE Working Group criteria (www.gradeworkinggroup.org).

The original 11 items were edited to 9 items (2 items combined and 1 item deleted) and clustered into 3 conceptual categories: clinical applicability, values, and implementability.

The original 3 response scales were modified to 2. The mandatory quality assessment scale asked appraisers to rate on the 7-point scale the overall quality of the item by considering whether the item criteria were addressed in the CPG and influenced the recommendations—for example, the extent to which data on the values and preferences of the various stakeholders were obtained and reported and extent to which these data were explicitly considered in formation of the recommendation.

The optional 7-point suitability for use scale is appropriate when a CPG is being considered for endorsement, adaptation, or implementation. This response scale considers whether the content of the criteria and its consequences for recommendations align with what would be expected in the context in which the CPG recommendations would be applied—for example, whether the potential users of a CPG perceive that the values and preferences of patients and policy makers collected and used to inform the CPG recommendations align with those in their own context. Appraisers are asked to rate the suitability for use in their setting/context.

In response to feedback, the 2 overall assessment questions (implementability and clinical credibility) were replaced by 2 new overall assessment questions to align with the AGREE II overall assessment items. The first new question (required) asked raters whether they would recommend the CPG for use in an appropriate context and the optional second new question asked raters whether they would recommend the CPG for use in their own context. A categorical response scale of yes, yes with modifications, and no is used to answer these assessment questions.

There was debate whether to integrate the new items into the existing AGREE II or have a separate AGREE-REX tool. A decision was made to create a separate tool to provide optimal flexibility to potential users. A resource to provide directions for use of the AGREE suite of tools has been written (M. C. Brouwers, PhD, unpublished data, 2020).

## Discussion

### Key Results and Interpretation

Overall, results of the study indicated that AGREE-REX is a usable, reliable, and valid tool to evaluate CPG recommendations. The AGREE-REX tool is a complement rather than an alternative to the AGREE II tool. The AGREE II tool focuses on the quality of the entire CPG process. The AGREE-REX tool focuses specifically on the quality of the CPG recommendations.

We believe that AGREE-REX will be a useful tool to evaluate CPG recommendations (single, bundle), differentiate among them, and identify those that are clinically credible and implementable for practicing health professionals and decision makers who use recommendations to inform clinical policy. Appraising a CPG with the AGREE II tool and the AGREE-REX tool may help provide information about the methodologic quality and the quality of the guideline recommendations. The appraisal step using both tools may help mitigate challenges in moving directly to costly and complex implementation commitments with CPGs that may lack rigor and suitability to the setting in which they are to be applied.

In addition to the evaluation version of the tool, we have created the AGREE-REX Reporting Checklist, which can be used to inform development and reporting standards. The criteria used for evaluation purposes are presented as quality concepts to be included and documented in the CPG as it is being developed and, moreover, to inform the development protocol. The checklist will help identify specific operational strategies to meet AGREE-REX quality criteria to incorporate from the outset. For example, the well-designed Evidence to Decision Framework reflects the utility of some of the AGREE-REX concepts.^35^ In addition, the checklist can help researchers prioritize when there is an absence of rigorous and feasible operational methods so efforts can be directed to address those gaps.

The recently released Clinical Practice Guidelines Applicability Evaluation (CPGAE-V1.0) also addresses this area. Designed to evaluate CPG applicability,^36^ the CPGAE-V1.0 has been used to assess traditional Chinese medicine guidelines but has not yet been tested by the international community, nor have its measurement properties been explored. Similarly, the recently released National Guideline Clearinghouse Extent of Adherence to Trustworthy Standards (NEATS instrument) is designed to measure CPG adherence to the Institute of Medicine standards for trustworthy guidelines.^37^ The methods of development and scope of these tools are different; nonetheless, investigating how the AGREE-REX tool and these tools complement each other may be a valuable area of inquiry.

Strengths of the AGREE-REX tool include the use of methodologic standards of measurement design in its development^29,32,33^; the use of multidisciplinary literature as a basis for the concepts underpinning AGREE-REX^30,31^; and its development by a multidisciplinary international research team and engagement of 322 internationally representative participants involved in CPGs. The participants reaffirmed the need for this tool, and their participation was vital to ensure that the resource was tailored to the needs of the international CPG communities.

### Limitations

This study has limitations. The measurement properties and usability surveys were performed with the penultimate draft version of the tool. Financial considerations prohibited the repetition of the studies to confirm that the changes made to the AGREE-REX tool were associated with improvements in measurement properties and usability. Nonetheless, we believe that decisions for modifications made were informed by evidence. Capturing information from in-the-field experiences on an ongoing basis will be essential in continuing to develop the evidence base to support use of the AGREE-REX tool. Additional supporting materials (eg, training tools) are being developed to improve interrater reliability of the tool. Another limitation is the criteria used to select the CPGs (<50 pages, English language only) and that the tool was applied to the whole set of recommendations in each report. Although the tool, and not the CPGs themselves, was the object of study, the criteria and unit of recommendation may affect the perceptions of the tool and its measurement properties. Continued application to a range of CPGs is required to better assess its generalizability.

## Conclusions

The results of this study suggest that AGREE-REX is a reliable, valid, and usable tool designed to evaluate CPG recommendations specifically. It is a complement to the AGREE II tool.

## Notes

Supplement. **eAppendix 1.** Draft AGREE-REX vs AGREE-REX Version 1 (V1) **eAppendix 2.** AGREE-REX: Recommendation Excellence User’s Guide
