# Supplementary material for: Development and Validation of a Tool to Assess the Quality of Clinical Practice Guideline Recommendations
Source: JAMA Netw Open. 2020 May 27;3(5):e205535. doi: 10.1001/jamanetworkopen.2020.5535 (PMC7254179; doi:10.1001/jamanetworkopen.2020.5535)
Supplement: Supplement. — eAppendix 1. Draft AGREE-REX vs AGREE-REX Version 1 (V1) eAppendix 2. AGREE-REX: Recommendation Excellence User’s Guide [file jamanetwopen-3-e205535-s001.pdf]

Supplementary Online Content

Brouwers MC, Spithoff K, Kerkvliet K, et al. Development and validation of a tool to assess the quality of clinical practice guideline recommendations. *JAMA Netw Open*. 2020;3(5):e205535. doi:10.1001/jamanetworkopen.2020.5535

eAppendix 1. Draft AGREE-REX vs. AGREE-REX Version 1 (V1)

eAppendix 2. AGREE-REX: Recommendation Excellence User's Guide

This supplementary material has been provided by the authors to give readers additional information about their work.

-

© 2020 Brouwers MC et al. *JAMA Network Open.*

Appendix 1. Draft AGREE-REX vs. AGREE-REX Version 1 (V1)

Draft AGREE-REX (used in testing)		AGREE-REX Version 1.0 (final version)	
**Domain**	**Items**	**Domain**	**Items**
Evidence Justification	1. Evidence	Clinical Applicability	1. Evidence<br>2. Applicability to Target Users<br>3. Applicability to Patients/Populations
Clinical Applicability Justification	2. Clinical Relevance<br>3. Relevance to Patients/Populations<br>4. Implementation Relevance		
Values Justification	5. Guideline Developer Values<br>6. Target User Values<br>7. Patient Population Values<br>8. Policy Values<br>9. Alignment of Values	Values and Preferences	4. Values and Preferences of Target Users<br>5. Values and Preferences of Patients/Populations<br>6. Values and Preferences of Policy/Decision-makers<br>7. Values and Preference of Guideline Developers
Feasibility Considerations	10. Local Applicability<br>11. Resources, Capacity and Tools	Implementability	8. Purpose<br>9. Local Application and Adoption
**Response Scales (R=required; O=optional)**<br>*1=strongly disagreement to 7=strongly agree*<br>1. Agreement that the item criteria were documented in the guideline (R)<br>2. Agreement that the item criteria were considered in formulating the recommendations (R)<br>3. Agreement that documentation and consideration of the item criteria were appropriate for the user's setting (O)		**Response Scales (R=required; O=optional)**<br>1. Overall quality of the item (R)<br>*1=lowest quality to 7=highest quality*<br>2. Agreement that the overall quality and interpretation of the item criteria are appropriate for the user's context (O)<br>*1=strongly disagree to 7=strongly agree*	
**Overall Quality Items**<br>*1=strongly disagree to 7=strongly agree*<br>1. Implementability of recommendations<br>2. Clinical credibility of recommendations		**Overall Quality Items (R=required; O=optional)**<br>*Yes, Yes With Modifications, No*<br>1. Recommend for use in the appropriate setting (R)<br>2. Recommend for use in my setting (O)	

© 2020 Brouwers MC et al. *JAMA Network Open.*

eAppendix 2

AGREE-REX:
Recommendation **EX**cellence

AGREE–REX Research Team 2019

© 2020 Brouwers MC et al. *JAMA Network Open.*

Published April 24, 2019
To access the most recent version of the AGREE-REX please visit the AGREE website at www.agreetrust.org.

© 2020 Brouwers MC et al. *JAMA Network Open.*

COPYRIGHT AND REPRODUCTION

This document is the product of an international collaboration. It may be reproduced and used for educational purposes, quality assurance programmes and critical appraisal of clinical practice guidelines. It may not be used for commercial purposes or product marketing. Offers of assistance in translation into other languages are welcome, provided they conform to the protocol set out by the AGREE Scientific office.

DISCLAIMER

The AGREE-REX is a tool designed to assess the quality of clinical practice guideline (CPG) recommendations. The authors do not take responsibility for the improper use of the AGREE-REX.

©2019

SUGGESTED CITATION FOR AGREE-REX PUBLICATION:

Manuscripts related to the AGREE-REX have been submitted to peer-reviewed journals for publication.
Citations will be added here when they are available.

SUGGESTED CITATION FOR AGREE-REX PDF VERSION:

AGREE-REX Research Team (2019). The Appraisal of Guidelines Research & Evaluation—Recommendation EXcellence (AGREE-REX) [Electronic version]. Retrieved <Month, Day, Year, from

FUNDING:

The development of the AGREE-REX tool was supported by the Canadian Institutes of Health Research.

FOR FURTHER INFORMATION ABOUT THE AGREE-REX DEVELOPMENT PROCESS,
RESEARCH TEAM, AND ADDITIONAL RESOURCES, PLEASE CONTACT:

AGREE Scientific Office, agree@mcmaster.ca

AGREE Enterprise Website, www.agreetrust.org

© 2020 Brouwers MC et al. *JAMA Network Open.*

AGREE-REX RESEARCH TEAM

Research Team Members:

Dr. M.C. Brouwers (Principal Investigator), McMaster University, Hamilton, Ontario and University of Ottawa, Ottawa, Ontario, Canada
Dr. P. Alonso-Coello, Iberoamerican Cochrane Centre, Barcelona, Spain
Dr. J.S. Burgers, Dutch College of General Practitioners, Utrecht, The Netherlands
Dr. F. Cluzeau, Global Health and Development Group, Imperial College London, UK
Dr. I.D. Florez, Universidad de Antioquia, Medellin, Colombia and McMaster University, Hamilton, Ontario, Canada
Dr. B. Fervers, Cancer et Environement, Centre Léon Bérard, France and Université de Lyon, Université Claude Bernard Lyon 1, Villeurbanne, France
Dr. A. Gagliardi, University Health Network, University of Toronto, Toronto, Ontario, Canada
Dr. I.D. Graham, Ottawa Hospital Research Institute, University of Ottawa, Ottawa, Ontario, Canada
Dr. J. Grimshaw, Ottawa Hospital Research Institute, University of Ottawa, Ottawa, Ontario, Canada
Dr. S.E. Hanna, McMaster University, Hamilton, Ontario, Canada
Dr. M. Kastner, North York General Hospital, Toronto, Ontario, Canada
Ms. K. Kerkvliet, McMaster University, Hamilton, Ontario, Canada
Dr. M.E. Kho, McMaster University, Hamilton, Ontario Canada
Dr. A. Qaseem, American College of Physicians, Philadelphia, Pennsylvania, USA
Dr. H. Schünemann, McMaster University, Hamilton, Ontario, Canada
Ms. K. Spithoff, McMaster University, Hamilton, Ontario, Canada
Dr. S. Straus, Li Ka Shing Knowledge Institute, St. Michael's Hospital, Toronto, Ontario, Canada

Acknowledgements:
Dr. O. Bhattacharyya, Women's College Hospital, University of Toronto, Toronto, Ontario, Canada
Dr. G.P. Browman, British Columbia Cancer Agency, Vancouver Island, Canada
Dr. P. Littlejohns, King's College London, London, UK
Ms. J. Makarski, McMaster University, Hamilton, Ontario, Canada
Dr. L. Zitzelsberger, Quebec, Canada

© 2020 Brouwers MC et al. *JAMA Network Open.*

OVERVIEW: AN INTRODUCTION TO THE AGREE-REX

BACKGROUND

Clinical practice guidelines are systematically developed statements informed by a systematic review of evidence and an assessment of the benefits and harms of alternative care options with the aim of optimizing patient care. They are informed by research evidence, values, and local/regional circumstances and inform decisions and judgements about health care at the clinical, management and policy levels[1,2].

The AGREE II has become an international methodological resource to inform guideline development, reporting, and evaluation[3]. Meeting rigorous methodological requirements is necessary but not sufficient to ensure that guideline recommendations are clinically credible or implementable. In response, and informed by research evidence and the participation of the international guideline community, the **AGREE-REX** (**A**ppraisal of **G**uidelines **RE**search and **E**valuation – **R**ecommendations **EX**cellence) was designed.

The AGREE-REX is a valid and reliable tool to assess the quality of guideline recommendations and a strategy to inform their development and reporting. The AGREE-REX aims to optimize the quality of guideline *recommendations,* defined as recommendations that are clinically credible, trustworthy, and implementable.

The AGREE-REX is a complement to the AGREE II.

The AGREE-REX addresses three factors that must be considered to ensure that guideline recommendations are of high quality. We define high quality recommendations as those that are clinically credible, trustworthy, and implementable. The three factors are:

- Clinical credibility of the recommendations based on the available evidence and its appropriateness for the target users, context, and patients/populations;
- Consideration of values of all relevant stakeholders in the formulation of the recommendations;
- Implementability of the recommendations.

The AGREE-REX can be applied to guidelines targeting any clinical or health topic and targeting any step in the health care continuum (health promotion, prevention, screening, diagnosis, treatment/intervention, and follow-up).

DEVELOPMENT OF THE AGREE-REX

Development of the AGREE-REX was led by an international team of practice guideline, knowledge translation, and methodology experts and researchers. A realist literature review was conducted to identify characteristics of guidelines that influence their implementability. The result of this work, the Guideline Implementability for Decision Excellence Model (GUIDE-M)[4,5], served as the basis for generating the AGREE-REX items. This was followed by a series of evaluations and refinements to establish the instrument's usability, reliability, and validity that involved hundreds of individuals in the guideline community world-wide.

AGREE-REX USERS

The AGREE-REX is intended for use by the following stakeholder groups:

- By **guideline developers** to evaluate existing guidelines to determine which are of adequate quality and appropriate for application or adaptation to their own context.
- By **guideline developers** to provide a methodological blueprint for de novo development that will yield high quality recommendations;

© 2020 Brouwers MC et al. *JAMA Network Open.*

- By **health care providers** who wish to undertake their own assessment to ensure guidelines recommendations are appropriate for adoption in their clinical setting;
- By **policy makers, health care administrators, program managers and professional organizations** to help them decide if guideline recommendations are appropriate to inform clinical practice strategies and policy design;
- By **researchers** who wish to assess the quality of guideline recommendations in a particular topic area;
- By **guideline database administrators** to assess the quality of guideline recommendations before inclusion in their database; and
- By **educators** to teach critical appraisal skills and core competencies in guideline recommendation development and reporting.
- By **any stakeholder** interested in supporting the improvement of practice guideline recommendation development, reporting, and evaluation.

AGREE-REX DOMAINS, ITEMS, AND CRITERIA

The AGREE-REX consists of nine items organized within three theoretical domains (Table 1), each focusing on a different factor that influences the quality of guideline recommendations. Each of the nine items has an operational definition and a list of specific criteria that characterize the concept. The number of criteria across the items ranges between 2 and 10.

Table 1. Domains and Items of the AGREE-REX

Domains	Items
1. Clinical Applicability	1. Evidence<br>2. Applicability to Target Users<br>3. Applicability to Patients/Populations
2. Values and Preferences	4. Values and Preferences of Target Users<br>5. Values and Preferences of Patients/Populations<br>6. Values and Preferences of Policy/Decision-Makers<br>7. Values and Preferences of Guideline Developers
3. Implementability	8. Purpose<br>9. Local Application and Adoption

HOW TO USE THE AGREE-REX: IN BRIEF

The AGREE-REX can be used for evaluation purposes to determine the degree to which guideline authors optimize the quality of the recommendations. It can also be used to inform guideline development and reporting requirements.

How To Use The AGREE-REX For Evaluation Purposes

The AGREE-REX includes two evaluation statements for each of the nine items. The first evaluation statement assesses whether the criteria that define each item were considered in formulating the recommendations and asks the user to rate the overall quality of this item. The second evaluation statement (optional) assesses the suitability or appropriateness of the guideline recommendations for a particular setting. Both items are answered using a 7-point response scale (1 [lowest quality] to 7 [highest quality]).

Depending on the needs of the user, the AGREE-REX can be applied to each individual guideline recommendation (or a prioritized set of individual recommendations), once to a group of guideline recommendations (e.g. a cluster of recommendations addressing a similar topic), or once to all guideline recommendations as a whole. Decisions about the level of AGREE-REX assessment should be based on the user's judgement.

© 2020 Brouwers MC et al. *JAMA Network Open.*

How To Use The AGREE-REX For Development and Reporting Purposes

The AGREE-REX item criteria can serve as a blue print by identifying the quality concepts that should be considered and incorporated into the development process and reported in the final guideline document. Determining any criteria that are not relevant to a particular guideline project should be done at the outset and a rationale for these decisions provided in the final guideline document.

How To Use The AGREE-REX With Other AGREE Tools

The AGREE-REX is a complement to the AGREE II (and the AGREE Global Rating Scale [GRS]). Whereas the AGREE II and AGREE GRS consider the entire guideline process, the AGREE-REX focuses specifically on the development and reporting of guideline recommendations. While there is no standard or required way to use the AGREE tools in combination, our recommendations are provided below:

- A combination of the AGREE Reporting Checklist and the AGREE-REX Reporting Checklist are recommended for use to support guideline development and reporting goals.
- Application of either the AGREE II or the AGREE GRS and the AGREE-REX are recommended to support evaluation goals.
- If the evaluation goals also include an interest in choosing or prioritize among candidate guidelines, the following strategies are proposed to make the process more efficient:
    1. Apply either the AGREE II or the AGREE GRS to narrow down a candidate list of guidelines that meet a minimum methodological threshold (e.g., a minimum of 50% on item or domain ratings) and then apply the AGREE-REX. This approach would be most appropriate if a user would not consider any guideline that did not meet minimum methodological development standards.
    2. Apply the AGREE-REX to narrow down the list of guidelines that meet a minimum recommendation quality threshold (e.g., a minimum of 50% of the overall AGREE-REX score) and then apply the AGREE II or the AGREE GRS. This approach would be appropriate for a user who would not consider any guideline that did not meet a minimum recommendation quality score.

ADDITIONAL RESOURCES

The AGREE-REX has been developed with the assumption that the user is familiar with basic evidence-based practice principles and the key components of a clinical practice guideline. If you are new to practice guidelines and would like more information, foundational resources include:

- Appraisal of Guidelines Research and Evidence (AGREE), www.agreetrust.org
- Grading of Recommendations Assessment, Development, and Evaluation (GRADE), www.gradeworkinggroup.org
- Guidelines International Network (G-I-N), www.g-i-n.net

Additional resources to assist with the application of the AGREE-REX will be made available on the AGREE Enterprise website at www.agreetrust.org as they are developed.

© 2020 Brouwers MC et al. *JAMA Network Open.*

REFERENCES

1. Woolf SH, Grol R, Hutchinson A, Eccles M, Grimshaw J. Clinical guidelines: potential benefits, limitations, and harms of clinical guidelines. BMJ 1999;318(7182):527-530.
2. Browman GP, Brouwers M, Fervers B, et al. Population-based cancer control and the role of guidelines-towards a “systems” approach, in Elwwod JM, Sutcliffe SB, (ed): Cancer control. Oxford, UK, Oxford University Press, 2010.
3. Brouwers MC, Kho ME, Browman GP, Burgers J, Cluzeau F, Feder G, Fervers B, Graham, ID, Grimshaw J, Hanna S, Littlejohns P, Makarski J, Zitzelsberger L on behalf of the AGREE Next Steps Consortium. AGREE II: Advancing guideline development, reporting and evaluation in healthcare. CMAJ 2010;182:E839-42..
4. Kastner M, Bhattacharyya O, Hayden L, Makarski J, Estey E, Durocher L, Chatterjee A, Perrier L, Graham ID, Straus S, Zwarenstein M, Brouwers M. Guideline uptake is influenced by six implementability domains for creating and communicating guidelines: a realist review. J Clin Epidemiol 2015;68(5):498-509.
5. Brouwers M, Makarski J, Kastner M, Hayden L, Bhattacharyya O, GUIDE-M Research Team. The Guideline Implementability Decision Excellence Model (GUIDE-M): a mixed methods approach to create an international resource to advance the practice guideline field. Implement Sci 2015;10:36.
6. Brouwers MC, Kerkvliet K, Spithoff K, AGREE Next Steps Consortium. The AGREE Reporting Checklist: a tool to improve reporting of clinical practice guidelines. BMJ 2016;352:i1152.

© 2020 Brouwers MC et al. *JAMA Network Open.*

INSTRUCTIONS: AGREE-REX

These instructions have been designed to assist users in the application of the AGREE-REX and should be reviewed before applying the tool.

HOW TO RATE

Review and Preparation

Before applying the AGREE-REX, a complete review of the guideline document and any additional supporting information within the document (e.g., tables, appendices) or published separately (e.g., methodological protocol) is required.

Level of Recommendation: Single, Cluster, or All

The AGREE-REX can be applied to assess the formation of a single (or prioritized) recommendation, a group or cluster of recommendations, or all the recommendations at once in a guideline document. A decision regarding level of recommendation should be made a priori, before evaluation begins and the rationale for the choice should be reported. Below is a list of considerations that can guide decisions about the level of recommendations to which the AGREE-REX should be applied.

Application of the AGREE-REX to a single recommendation or group of recommendations is most appropriate when:

- The AGREE-REX user believes that quality may vary between recommendations in the guideline being assessed; or,
- Only selected recommendations (or a single recommendation) are of interest and are being considered for adaptation, endorsement, or implementation.

Application of the AGREE-REX to all the guidelines recommendations is most appropriate when:

- The AGREE-REX user believes that quality is consistent between recommendations in the guideline being assessed; or,
- All guideline recommendations are of interest and are being considered for adaptation, endorsement or implementation; or,
- Resource and time constraints make it impractical to evaluate each recommendation (or group of recommendations) separately.

Rating Scale and Assessment Process

The AGREE-REX includes two evaluation statements for each item: one to assess overall quality (required) and one to asses suitability for use (optional). It also includes two overall assessment statements to apply to the whole guideline (again, one required and one optional).

Quality Assessment: Rate the overall quality of this item.

1<br>Lowest quality	2	3	4	5	6	7<br>Highest quality

This evaluation statement should be applied to determine whether criteria to optimize clinically credibility, trustworthiness, and implementability were considered in formulating the recommendations. All items are rated using a 7-point scale (1 [lowest quality] to 7 [highest quality]).

- A score of 1 should be given if there is no information that is relevant to the AGREE-REX item's criteria or the item's criteria were not considered in the formulation of the guideline recommendations.

© 2020 Brouwers MC et al. *JAMA Network Open.*

- A score of 7 should be given if all the item's criteria have been carefully and thoroughly considered in the formulation of the recommendation(s).
- A score between 2 and 6 should be given when some but not all of the item's criteria are considered in the formulation of the recommendation(s) and/or the link between the criteria and the recommendations is not optimal.
- The appraiser should provide their reasoning for the score in the comments box provided. This is useful for discussion with other appraisers.

Suitability for Use (Optional): The overall quality and interpretation of the item criteria are appropriate for my context.

**1** Strongly Disagree	**2**	**3**	**4**	**5**	**6**	**7** Strongly Agree

This evaluation statement is optional and can be applied to the items if the goal of the evaluation is also to determine whether or not the guideline recommendations are appropriate for use in a particular setting. All items are rated using a 7-point scale (1 [strongly disagree] to 7 [strongly agree]).

- A score of 1 should be given when there is no information that is relevant to the AGREE-REX item's criteria or and interpretation of the item's criteria are not appropriate for the context in which the appraiser intends to use the guideline recommendations.
- A score of 7 should be given if the quality is excellent and the interpretation of the item's criteria are appropriate for the context in which the guideline will be used.
- A score between 2 and 6 should be given if some but not all of the interpretations of the item's criteria associated with the recommendation are appropriate for the context in which the guideline will be used.
- The appraiser should provide their reasoning for the score in the comments box provided.

Overall Assessment Statements:

The overall assessment statements require the user to make a judgement about whether the appraiser would recommend the guideline recommendations for use 1. in the appropriate context, and, if applicable, 2. in the appraiser's context. The appraiser has three answer options: yes, yes with modifications, or no.

1. I would recommend these guideline recommendations for use **in the appropriate context**.

Yes
Yes, with modifications
No

2. I would recommend these guideline recommendations for use **in my context (optional)**.

Yes
Yes, with modifications
No

© 2020 Brouwers MC et al. *JAMA Network Open.*

Calculating AGREE-REX Scores

AGREE-REX results can be calculated and reported in various ways, including as item scores, domain scores, or an overall score. In addition, users must decide whether the scores will be calculated using individual scores from multiple appraisers or if appraisers will be required to reach consensus on scores.

Using Individual Appraisers' Scores vs. Consensus Scores

Using individual scores from multiple appraisers to calculate AGREE-REX scores preserves the variability and different perspectives of the appraisers. This approach is used when appraisers do not meet to discuss their scores. The reliability assessment of the tool was completed on its penultimate version and based on these data, five independent appraisers should be recruited if a consensus process will not be undertaken.

When there is an opportunity for multiple appraisers to meet to discuss scores, users may choose to use a consensus approach to reach agreement about AGREE-REX item scores. This method is also appropriate. The consensus score should be then applied to the calculation described below.

Item Scores, Domain Scores, and Overall Score

Item scores
AGREE-REX items scores can be calculated by averaging the individual appraisers' scores (i.e., calculating the mean) on the 7-point scale (1=strongly disagree; 7=strongly agree) for each of the nine items. If a consensus approach is used to determine scores, then the consensus scores are the item scores. Advantages of reporting item scores are that no assumptions need to be made about the weighting or relative importance of the items, and it allows users to make observations or comparisons at the item level.

Domain scores
AGREE-REX domain scores can be calculated by adding all the scores of the individual items in a domain (the sum of the item scores is referred to as the "obtained score" in the formula below) and by scaling the total as a percentage of the maximum possible score. If item scores are determined by consensus, the same formula can be used. Reporting domain scores allows users to make observations and comparisons based on domain themes (i.e., clinical applicability, values, and implementability). The limitation of this method is that the clustering of the nine items into the three domains is based on the face validity of the cluster, and not empirical evidence. In addition, there is no empirical evidence available to determine the weighting or relative importance of the items within the domains; in the formula below, all items are given equal weighting within a domain.

Example:

If five appraisers give the following sores for Domain 1 (Clinical Applicability):

	Item 1	Item 2	Item 3	**Total**
Appraiser 1	5	6	4	**15**
Appraiser 2	6	6	3	**15**
Appraiser 3	4	7	5	**16**
Appraiser 4	5	5	4	**14**
Appraiser 5	4	6	4	**14**

© 2020 Brouwers MC et al. *JAMA Network Open.*

Total	24	30	20	74

Maximum possible score = 7 (highest quality) x 3 (items) x 5 (appraisers) = 105
Minimum possible score = 1 (lowest quality) x 3 (items) x 5 (appraisers) = 15

The scaled domain score will be:

$$\frac{\text{Obtained score} - \text{Minimum possible score}}{\text{Maximum possible score} - \text{Minimum possible score}}$$

$$\frac{74 - 15}{105 - 15} \text{ X } 100 = \frac{59}{90} \text{ X } 100 = 0.6556 \text{ x } 100 = 66\%$$

If multiple appraisers reach consensus on scores for Domain 1 (Clinical Applicability):

	Item 1	Item 2	Item 3	**Total**
Consensus Score	4	6	4	**14**

$$\frac{\text{Obtained consensus score} - \text{Minimum possible score}}{\text{Maximum possible score} - \text{Minimum possible score}}$$

$$\frac{14 - 3}{21 - 3} \text{ X } 100 = \frac{11}{18} \text{ X } 100 = 0.6111 \text{ x } 100 = 61\%$$

Overall score
An AGREE-REX overall score can be calculated by adding all nine item scores and using the formula above to scale the total as a percentage of the maximum possible scale. If item scores are determined by consensus, the same formula can be used. Reporting an overall score provides a simple way to describe the quality of guideline recommendations overall and to compare between multiple guidelines. However, an overall score on its own does not provide precise information about the particular strengths and weaknesses of the guideline recommendations. In addition, an overall score assigns equal weighting to each of the nine items, but there is no evidence available to determine the relative importance of the items in determining the quality of guideline recommendations.

Interpreting AGREE-REX Scores

At present, there are no empirical data to link specific quality scores (item scores, domain scores or overall scores) with specific implementation outcomes (e.g., speed of adoption, spread of adoption) or specific clinical outcomes; this makes selection of quality thresholds to differentiate between high, moderate, or low quality guideline recommendations a challenge. In the absence of these data, we provide examples of approaches that can be used to set quality thresholds:

- Users could perform a tertile split of the overall score (or domain scores or overall score) of the candidate guidelines being considered and classify documents as being higher quality, moderate quality, or lower quality.
- Users may determine threshold scores through consensus among stakeholders or appraisers. For

© 2020 Brouwers MC et al. *JAMA Network Open.*

example, guidelines with overall scores >70% may be defined as high quality, those with overall quality scores <30% lower quality, and all others moderate quality.
- Users might value one item or domain over the others for their decision-making purposes and create thresholds based on that item or domain.
- Users may use AGREE-REX Scores as a continuous variable and conduct modelling exercises to determine what AGREE-REX scores predict certain outcomes and use that score as the threshold.

Any decisions about how to define minimum thresholds for quality or applicability should be made by a panel of all relevant stakeholders before beginning the AGREE-REX appraisals. Decisions should be guided by the context in which the practice guideline is to be used and by evaluating the importance of the different items and criteria in that context. For example, stakeholders can use scores to compare practice guidelines documents and identify limitations of the guidance being considered, or to select high quality practice guidelines to implement.

ADDITIONAL ASSESSMENT CONSIDERATIONS

Clarity of Presentation

When evaluating each AGREE-REX item, the following questions should also be considered:
- Is the information well written (i.e., clear and concise)?
- Is the information easy to find in the guideline?
- Does the guideline provide the user with an appropriate level of transparency?

Applicability of AGREE-REX Items

On occasion, some AGREE-REX items may not be applicable to the particular guideline under review. There are different strategies to manage this situation, including skipping that item in the assessment process or rating the item as 1 (absence of information) and providing context about the score. Regardless of the strategy chosen, decisions should be made in advance and described in an explicit manner. As a principle, excluding items from the appraisal process is discouraged.

User's Judgement in Appraising

How the AGREE-REX is applied and the actual evaluation process requires a level of judgement. Be explicit about choices and provide a rationale for the decisions made.

AGREE-REX TOOL

© 2020 Brouwers MC et al. *JAMA Network Open.*

Item 1. Evidence

In order for recommendations to be of high quality, they should be based on a thorough review of the quality and results of the available evidence. In formulating the recommendations and developing the guideline, the following issues should be addressed:

Criteria[a]**:**

- The guideline assesses any risk of bias related to the study designs of the supporting evidence.
- The guideline describes the consistency of the results (i.e., similarity of results across studies).
- The guideline addresses the directness of the evidence (i.e., addresses the exact interventions, populations and outcomes of interest) to the clinical/health problem.
- The guideline indicates the precision of the results (e.g., width of confidence intervals of individual studies or meta-analyses).
- The guideline describes the magnitude of the benefits and harms.
- The guideline assesses the likelihood of publication bias.
- The guideline addresses the possibility of confounding factors (if applicable).
- The guideline indicates the dose-response gradient (if applicable).

[a] Informed by GRADE Working Group criteria (www.gradeworkinggroup.org)

Quality Assessment:
Rate the overall quality of this item.

1<br>Lowest quality	2	3	4	5	6	7<br>Highest quality

Comments

Suitability for use (optional):
The overall quality and interpretation of the item criteria are appropriate for my context.

1<br>Strongly disagree	2	3	4	5	6	7<br>Strongly agree

Comments

© 2020 Brouwers MC et al. *JAMA Network Open.*

Item 2. Applicability to Target Users

This item evaluates the degree to which the recommendations are applicable to the guideline's target users' practice context. In formulating the recommendations and developing the guideline, the following issues should be addressed:

Criteria:

- The guideline addresses a clinical/health problem that is relevant to the intended target user(s).
- There is an alignment between
    - target user's scope of practice and targeted patients/populations.
    - target user's scope of practice and recommended actions.
    - the direction of the recommendations (i.e., in favour of or against a particular action) and the trade-offs between harms and benefits.
    - the definitiveness or strength of the recommendations and the trade-offs between harms and benefits.

Quality Assessment:
Rate the overall quality of this item.

1<br>Lowest quality	2	3	4	5	6	7<br>Highest quality

Comments

Suitability for use (optional):
The overall quality and interpretation of the item criteria are appropriate for my context.

1<br>Strongly disagree	2	3	4	5	6	7<br>Strongly agree

Comments

© 2020 Brouwers MC et al. *JAMA Network Open.*

Item 3. Applicability to Patients/Populations

This item assesses the extent to which the **anticipated outcomes** of the recommended action are relevant for, and valued by, the intended patients/populations. In formulating the recommendations and developing the guideline, the following issues should be addressed:

Criteria:

- The guideline includes outcomes that are relevant to the targeted patients/populations. These outcomes are often referred to as patient important outcomes, patient centered outcomes, patient reported outcomes, or patient experience.
    - Relevant outcomes were considered in the development of the evidence base.
    - Recommended actions have the potential to impact outcomes relevant to patients/populations (e.g., improve desirable patient-relevant outcomes, mitigate undesirable patient-relevant outcomes).
- The guideline reports how the importance of outcomes to patients was determined.
- The guideline describes how to tailor recommendations for application to individual (or subsets of) patients or populations (e.g., based on age, sex, ethnicity, comorbidities).

Quality Assessment:
Rate the overall quality of this item.

**1** Lowest quality	**2**	**3**	**4**	**5**	**6**	**7** Highest quality

Comments

Suitability for use (optional):
The overall quality and interpretation of the item criteria are appropriate for my context.

**1** Strongly disagree	**2**	**3**	**4**	**5**	**6**	**7** Strongly agree

Comments

© 2020 Brouwers MC et al. *JAMA Network Open.*

Item 4. Values and Preferences of Target Users

Values and preferences of target users refers to the relative importance that the target users of the guidelines (e.g., health care providers, policy-makers, administrators) place on the outcomes of interest (e.g., survival, adverse effects, quality of life, cost, convenience). Target user values and preferences are important to consider during the guideline development process because they influence whether the recommendations are acceptable and adopted into practice. In formulating the recommendations and developing the guideline, the following issues should be addressed:

Criteria

- Values and preferences of guideline target users, as it relates to the recommended actions, have been sought and considered.
- Factors related to target user acceptability of the recommended actions have been considered (e.g., the acceptability of learning new clinical skills or the need to adapt current routine).
- The guideline differentiates between recommended actions for which clinical flexibility and individual patient tailoring is more appropriate in the decision-making process and those for which it is less appropriate.
- The guideline describes the range of recommended actions that are acceptable to the clinical community, including the preferred option (if relevant), and describing why it is the preferred choice.

Quality Assessment:
Rate the overall quality of this item.

**1**<br>Lowest quality	**2**	**3**	**4**	**5**	**6**	**7**<br>Highest quality

Comments

Suitability for use (optional):
The overall quality and interpretation of the item criteria are appropriate for my context.

**1**<br>Strongly disagree	**2**	**3**	**4**	**5**	**6**	**7**<br>Strongly agree

Comments

© 2020 Brouwers MC et al. *JAMA Network Open.*

Item 5. Values and Preferences of Patients/Populations

Values and preferences of patients/populations refers to the relative importance that the recipients of the recommended actions place on the outcomes of interest (e.g., survival, adverse effects, quality of life, cost, convenience). Patient or population values and preferences are important to consider during the guideline development process because they influence whether the recommendations are acceptable and adopted into practice. In formulating the recommendations and developing the guideline, the following issues should be addressed:

Criteria:

- Values and preferences of the target population (including patients, family and caregivers, if appropriate) have been sought and considered.
- Factors related to patient/population acceptability of the recommended actions have been considered (e.g., motivation, ability to achieve outcomes, expectations, perceived effectiveness).
- The guideline differentiates between recommended actions for which patient choice and/or values are likely to play a large part in the decision-making process and those for which they are likely to play a small role.
- The guideline states whether tools to assist in patient decision-making would be beneficial.

Quality Assessment:
Rate the overall quality of this item.

**1**<br>Lowest quality	**2**	**3**	**4**	**5**	**6**	**7**<br>Highest quality

Comments

Suitability for use (optional):
The overall quality and interpretation of the item criteria are appropriate for my context.

**1**<br>Strongly disagree	**2**	**3**	**4**	**5**	**6**	**7**<br>Strongly agree

Comments

© 2020 Brouwers MC et al. *JAMA Network Open.*

Item 6. Values and Preferences of Policy/Decision-Makers

Values and preferences of policy/decision-makers refers to the relative importance that policy stakeholders place on the outcomes of interest (e.g., survival, adverse effects, quality of life, cost, convenience). The values and preferences of policy stakeholders can affect the implementation of guideline recommendations in the health care system (e.g., provision of resources or funding to support the recommended actions). In formulating the recommendations and developing the guideline, the following issues should be addressed:

Criteria:

- Information about the needs of policy and decision-makers has been sought and considered in the formulation of the recommendations.
- The impact of the recommendations on policy and system-level decision-making has been considered in the formulation of the recommendations.
- The impact of the recommendations on health equities has been considered in the formulation of the recommendations.
- The guideline describes where changes to policy should be made to align with the recommendations.

Quality Assessment:
Rate the overall quality of this item.

**1**<br>Lowest quality	**2**	**3**	**4**	**5**	**6**	**7**<br>Highest quality

Comments

Suitability for use (optional):
The overall quality and interpretation of the item criteria are appropriate for my context.

**1**<br>Strongly disagree	**2**	**3**	**4**	**5**	**6**	**7**<br>Strongly agree

Comments

© 2020 Brouwers MC et al. *JAMA Network Open.*

Item 7. Values and Preferences of Guideline Developers

Values and preferences of guideline developers refers to the relative importance that developers place on the outcomes of interest (e.g., survival, adverse effects, quality of life, cost, convenience). Guideline developer values can influence the selection of outcomes of interest, the choice of guideline development methods, the approach to integrating varying stakeholder perspectives, and the interpretation of the balance between benefits and harms. In formulating the recommendations and developing the guideline, the following issues should be addressed:

Criteria:

- There is a clear description of the values and preferences that guideline developers brought to the development process.
- There is a clear description of how guideline developer values and preferences influenced their interpretation of the balance between benefits and harms.
- The method used to integrate values and preferences, including when they differ between stakeholders (e.g., target users, patients/population, policymakers), is described.

Quality Assessment:
Rate the overall quality of this item.

**1**<br>Lowest quality	**2**	**3**	**4**	**5**	**6**	**7**<br>Highest quality

Comments

Suitability for use (optional):
The overall quality and interpretation of the item criteria are appropriate for my context.

**1**<br>Strongly disagree	**2**	**3**	**4**	**5**	**6**	**7**<br>Strongly agree

Comments

© 2020 Brouwers MC et al. *JAMA Network Open.*

Item 8. Purpose

Practice guidelines can be developed to achieve several implementation goals, such as to influence health care decisions, to promote discussion in the clinical encounter, to provide rationale to create or refine clinical policy, or to identify actions that reflect clinical or population health goals. In formulating the recommendations and developing the guideline, the following issues should be addressed:

Criteria:

- The guideline recommendations align with the implementation goals of the guideline (e.g., for advocacy, policy change, etc.).
- The anticipated impacts of recommendation adoption on individuals (e.g., patients, populations, target users), organizations, and/or systems are described.

Quality Assessment:
Rate the overall quality of this item.

**1** Lowest quality	**2**	**3**	**4**	**5**	**6**	**7** Highest quality

Comments

Suitability for use (optional):
The overall quality and interpretation of the item criteria are appropriate for my context.

**1** Strongly disagree	**2**	**3**	**4**	**5**	**6**	**7** Strongly agree

Comments

© 2020 Brouwers MC et al. *JAMA Network Open.*

Item 9. Local Application and Adoption

This item assesses the suitability of the guideline recommendations for the setting, patients/population, and/or the health care system in which they are being implemented. Guidelines that include advice or tools and resources to facilitate the implementation of the recommendations are easier to adopt in practice. In formulating the recommendations and developing the guideline, the following issues should be addressed:

Criteria:

- The guideline describes the types and degree of change required from current practice.
- The guideline differentiates between recommendations for which local adaptation may be more or less relevant.
- The guideline articulates relevant factors important to its successful dissemination.
- The guideline developers considered the issues that can influence the adoption of the recommendations and provided tools and/or advice for guideline implementers related to:
    - How to tailor recommendations for the local setting.
    - Resource considerations needed to implement the recommendations (e.g., human resources, equipment) and their associated costs.
    - Economic analysis (e.g., cost-effectiveness or cost-utility) of recommended actions (if appropriate).
    - Competencies and/or training of personnel required to implement the recommended actions.
    - Data required to implement and monitor the adoption of recommended actions.
    - Strategies to overcome barriers related to provider acceptability and/or patient/population and/or policy acceptability of the recommended actions.
    - Criteria that can be used to measure recommendation implementation and quality improvement.

Quality Assessment:
Rate the overall quality of this item.

1<br>Lowest quality	2	3	4	5	6	7<br>Highest quality

Comments

Suitability for use (optional):
The overall quality and interpretation of the item criteria are appropriate for my context.

1<br>Strongly disagree	2	3	4	5	6	7<br>Strongly agree

Comments

© 2020 Brouwers MC et al. *JAMA Network Open.*

OVERALL

1. I would recommend these guideline recommendations for use **in the appropriate context**.

Yes	
Yes, with modifications	
No	

Comments

2. I would recommend these guideline recommendations for use **in my context** (optional).

Yes	
Yes, with modifications	
No	

Comments

© 2020 Brouwers MC et al. *JAMA Network Open.*
